# Evaluation of the effect of different diets applied to breastfeeding mothers on the composition and quantity of human milk

**DOI:** 10.1038/s41430-025-01588-z

**Published:** 2025-03-10

**Authors:** Canel Öner Sayar, Sabiha Zeynep Aydenk Köseoğlu

**Affiliations:** 1 Department of Nutrition and Dietetics, School of Health Sciences, Istanbul Medipol University, Istanbul, Turkey; 2 Department of Nutrition and Dietetics, School of Health Sciences, Bezmiâlem Vakif University, Istanbul, Turkey

**Keywords:** Proteins, Nutrition, Clinical trial design, Endocrine system and metabolic diseases

## Abstract

**Introduction:**

This study aimed to elucidate the relationship between maternal nutrition and the quantity and composition of breast milk.

**Methods:**

All mothers were administered sequentially, with each lasting one week, a healthy nutrition diet, a carbohydrate-rich diet, and a protein-rich diet.

**Results:**

Compared to the healthy nutrition diet, a statistically significant increase was observed in the levels of glutamic acid, serine, glycine, histidine, tyrosine, valine, isoleucine, leucine, and lysine in milk following the carbohydrate-rich diet (*p* < 0.05). In contrast to the healthy nutrition diet, the lactose content of milk decreased after the carbohydrate-rich diet and increased after the protein-rich diet (*p* < 0.05). Following both carbohydrate-rich and protein-rich diets, a reduction in riboflavin content in milk was observed compared to the healthy nutrition diet (*p* < 0.05). After the protein-rich diet, an increase in milk quantity was observed compared with the carbohydrate-rich diet (G1, *p* = 0.006; G2, *p* = 0.001). A negative correlation was found between the mothers’ body weight in the third week and the amount of amino acids in their milk (*r* = -0.270, *p* = 0.037).

**Conclusions:**

The results of this study indicated that the nutrient composition and quantity of human milk are influenced by maternal nutrition.

## Background

Ensuring healthy nutrition during lactation is crucial to the well-being of both mothers and infants. Healthy nutrition among mothers is a significant factor influencing the volume [1] and composition of human milk [2]. Therefore, maternal diet during this period may even impact the health of infants in adulthood [3, 4].

The total quantity of milk produced is a crucial component that influences lactation performance [5]. One factor affecting milk quantity is insufficient nutrition of the mother or the implementation of restrictive diets, which can negatively affect milk production [6]. Maternal daily energy intake below 1500 kcal can lead to a decrease in milk volume and a sense of fatigue in the mother [7]. Existing studies on the effects of adequate protein intake on milk production are outdated and inadequate. Some studies have suggested that increasing protein intake in mothers’ diets has a positive impact on both milk production and the amount of milk consumed by infants [8, 9]. During the first eight weeks after childbirth, lactating women lose an average of 700 mL of water daily, considering that 87% of human milk consists of water. Consequently, the daily water requirements of mothers increase during the breastfeeding period [10]. However, there is insufficient evidence regarding whether consuming more water than necessary has a positive effect on milk production [11].

Several studies have investigated the composition of human milk. Research on the effects of nursing mothers’ diets on carbohydrates in human milk remains uncertain in terms of the impact of different diets. It has been shown that the total carbohydrate content of milk is not significantly affected by the total energy intake [12] a high-protein diet [13] or a diet rich or low in carbohydrates and fats [14] This indicates that the diet of nursing mothers partially influences their amino acid profile and milk protein levels [5, 15]. In one study, a significant positive relationship, particularly at the protein level, was found between the amounts of protein, fat, carbohydrates, and energy obtained from the mother’s diet and the levels of macronutrients in milk [16]. When the mother’s diet was low in protein, a decrease in non-protein nitrogen and total free amino acid content in milk was observed, and the ratio of whey protein to casein was positively correlated with protein intake [17]. A study conducted in Sweden demonstrated that mothers who consumed a high-protein diet had a higher protein content in their milk than mothers who consumed a low-protein diet [13]. The mother’s body regularly stores vitamin B_2_ and utilizes it during breastfeeding. However, it has been reported that the vitamin B_2_ requirement of breastfeeding mothers may increase and that the concentration of vitamin B_2_ in their milk may decrease if they do not consume an adequate amount of vitamin B_2_ [18].

There are studies worldwide investigating the impact of maternal nutrition on human milk. However, most of these studies were dated, and there is a need for further research. Hence, the objective of this study was to determine the impact of the nutritional intake of lactating mothers based on different macronutrient distributions on the quantity of expressed breast milk as well as its effects on lactose, B_2_ vitamin, and certain amino acid components.

## Methods

### Ethical approval and informed consent

This study was conducted on lactating mothers aged 4–24 weeks who presented to the pediatric department of a private hospital located in the district of Istanbul, both in vivo and in vitro. The study was conducted between September 1, 2021, and September 1, 2022, after obtaining the necessary permissions from the hospital and ethical board approval from the Medipol University Non-Interventional Clinical Ethics Committee (Number: 10840098-772.02-2857, date: 17/06/2021). All methods used in this study were conducted in accordance with the relevant guidelines and regulations. For one month, lactating mothers who presented to the pediatric department of a private hospital located in the district of Istanbul constituted the study population. Informed consent was obtained from all the participants.

### Participant recruitment

The sample group was selected from among voluntary mothers who were included in this universe and met the specified study criteria. The eligibility criteria for participation included being a Turkish citizen, aged between 24 and 35 years, in the lactation periods of 4–7 or 13–19 weeks at the beginning of the study, feeding the infant only breast milk, expressing breast milk using a breast pump, not implementing a specific dietary program during the lactation period, refraining from engaging in heavy or regular physical activity, not undergoing treatment for any illness, not employed during the research period, having given birth at term, avoiding the use of herbal or chemical supplements that may affect milk production, not taking medications for any chronic conditions, abstaining from smoking and alcohol consumption, not using formula milk or introducing complementary feeding, the infant does not having a condition preventing breastfeeding (such as heart disease), and voluntary participation in the study. The exclusion criteria for constructing the sample were illness during the data collection period, starting medication during the study, not adhering to the prescribed nutrition programs, not attending routine meetings, and voluntarily leaving the study.

Considering that there may be periodic changes in the amount of breast milk, the mothers were divided into four groups for the study. The study was conducted with a total of 72 mothers, comprising 18 mothers aged 24–29 in the 13–24 weeks lactation period, 18 mothers aged 30–35 in the 13–24 weeks lactation period, 18 mothers aged 24–29 in the 4–12 weeks lactation period and 18 mothers aged 30–35 in the 4–12 weeks lactation period. Based on the results of the post-power analysis conducted on milk quantity and milk components, with an alpha level of 0.05, power of 0.85, and effect size of 0.45, the power of the study was determined to be 85% when evaluated based on milk components, and 99.9% when assessed using data obtained from milk quantity.

### Diets

In this study, three different nutrition programs were sequentially applied to mothers: a healthy nutrition diet in the first week, a carbohydrate-rich diet in the second week, and a protein-rich diet in the third week. The energy content of the nutrition programs was set at 2200 calories, based on the caloric recommendations for lactating mothers in the Dietary Guidelines for Turkey [19]. In the healthy nutrition diet, the energy distribution was planned as 55–60% carbohydrates, 10–15% protein, and 25–30% fat. Based on the information obtained from previous studies [14, 20, 21] the ratios in the carbohydrate-rich diet were determined as follows: 65% carbohydrates, 15% protein, and 20% fat, whereas in the protein-rich diet, the distribution was adjusted to 50% carbohydrates, 25% protein, and 25% fat. To alter the macronutrient composition of the diets, the amounts of foods in the healthy nutrition diet were adjusted. The same foods were used in all diets, and no different foods were introduced. To ensure that the mothers did not lose adherence to the diet process and because a one-week diet period was deemed sufficient to impact the quantity and composition of breast milk, no ‘washout’ period was implemented between the three diets. Participants’ adherence to the nutrition programs was regularly assessed every week through dietary records, with one record taken on a weekend and the other on a weekday.

### Data collection

Anthropometric measurements were taken at the beginning of the study and after each dietary program (at the end of each week). Mothers’ body weights were measured using TANITA BC-730 brand bioelectrical impedance analysis (BIA). Body composition analysis of mothers was performed after they had visited the restroom, without engaging in physical activity, and at least 2–3 hours after their last meal and fluid intake [22]. Mothers’ height was measured using a stadiometer. Waist and hip circumferences were measured according to measurement principles, and the waist-to-hip ratio was calculated [23].

In accordance with the baby care pamphlet provided by the hospital, the mothers were educated on proper breastfeeding techniques. The mothers received training on this subject at the beginning of the study and at the beginning of each week. At the conclusion of each week, mothers visited the researcher at least two hours after breastfeeding their infants between 09:00 and 12:00. The researcher conducted a 20-minute milking session on the mothers using a Philips Avent SCF395/11 Advanced Single Electric Breast Pump. The quantity of milk (preferably from the right breast) was recorded in milliliters and provided to the mothers in human milk storage pouches.

The estimated daily total expressed milk volume was determined by multiplying the quantity of milk pumped from one breast by the mother’s breastfeeding quantity during the day (average of the last 3 days’ meals). During milking, 10 mL of human milk was sampled from the middle milk and transferred to sterile 15 mL Falcon tubes. The collected human milk samples were stored at −20°C in a deep freezer at Sabri Ülker Food and Nutrition R&D Center, Istanbul Sabahattin Zaim University, until the analysis stage, and were analyzed within six months. The analyses included the examination of lactose, aspartic acid, glutamic acid, serine, arginine, histidine, glycine, threonine, alanine, proline, tyrosine, valine, methionine, leucine, isoleucine, phenylalanine, lysine, tryptophan, and vitamin B_2_ in the milk samples.

### Milk analysis

The analysis of amino acids was conducted using a method modified by Gheshlaghi et al. [24]. The samples were derivatized using a phenyl isocyanate solution and subsequently dried. Subsequently, they were dissolved in a 0.02 M ammonium sulfate solution. After filtration, the solution was analyzed using a high-performance liquid chromatography (HPLC) system with an Agilent Eclipse XDB-C18 column (dimensions: 5 µm, 4×6×150 mm) connected to a Shimadzu UFLC20A. The mobile phase was prepared using a sodium phosphate buffer solution at different ratios and acetonitrile at pH 6.9. The detection was performed using a UV detector at a wavelength of 254 nm.

The analysis method for vitamin B_2_ in milk samples relied on measuring the peak area in a fluorescent (FI) detector using high-performance liquid chromatography (HPLC) after the sample in 0.1 N hydrochloric acid was autoclaved at 121°C for 30 min and then subjected to enzymatic incubation at 45°C [25]. The HPLC conditions were as follows: mobile phase, Water: Methanol (75:25); Detector: FI, Wavelength: Excitation, 445 nm; emission, 525 nm; Injection Volume: 20–50 µl, Flow Rate, 1 ml/minute; Analysis Time, 20 min. The column used was An Agilent Eclipse XCD-C18, 5 μm, 4.6×150 mm.

The lactose content in human milk was determined by dissolving the milk sample in an appropriate solvent mixture, followed by centrifugation or passage through a cartridge, and measurement of the peak area in a refractive index (RI) detector using HPLC [26]. HPLC Conditions: Detector: RI, Mobile Phase: Acetonitrile: Water, 80:20, v/v, 0.22 µm membrane filter, Column Temperature: 25–30°C, Injection Volume: 10 µL, Flow Rate: 2 ml/minute, Analysis Time: 18 min. An analytical normal-phase column was used (Merck LiChroCART NH2, 5 µm, 4.6×250 mm).

### Statistical analysis

Statistical analysis of the data obtained in this study was conducted using IBM SPSS Statistics 22. Descriptive statistical methods, including mean, standard deviation, and frequency, were used for data evaluation. The normal distribution appropriateness of the data was assessed through histogram, Q-Q plots, Kolmogorov–Smirnov, and Shapiro–Wilks tests. In cases where parameters exhibited a normal distribution, Repeated Measures Analysis of Variance (post hoc Bonferroni Test) was used for within-group comparisons. For parameters not showing a normal distribution, the Friedman Test (post-hoc Wilcoxon signed-rank test) was applied for within-group comparisons. Dunn–Bonferroni test was used for multiple comparisons. Pearson correlation analysis was used to examine the relationships between parameters exhibiting a normal distribution, while Spearman’s rho correlation analysis was applied for parameters that did not conform to a normal distribution. Statistical significance was set at *p* < 0.05.

## Results

G1 represents mothers aged 24–29 in the 13–24 weeks lactation period, G2 represents mothers aged 30–35 in the 13–24 weeks lactation period, G3 represents mothers aged 24–29 in the 4–12 weeks lactation period, and G4 represents mothers aged 30–35 in the 4–12 weeks lactation period. The ages of the mothers range from 24 to 35, with an average age of 29.51 ± 3.64 years, and an average height of 161.30 ± 5.5 cm. In G1, 66.7% of the participants in G2, 55.5%; G3, 72.2%; and G4, 66.7% were high school graduates. Among all the participants, 68% were homemakers and did not engage in employment.

In G1 and G2, a statistically significant decrease in the average milk quantity was observed in the second week compared with that in the first week (*p* = 0.037 and *p* = 0.001, respectively). Furthermore, in G1 and G2, the increase in milk quantity observed in the third week compared to that in the second week was statistically significant (*p* = 0.006 and *p* = 0.001, respectively) (Table 1).

**Table 1:** Comparison of weekly average human milk quantities based on the given diets.

Total Milk Quantity (ml)	G1 (*n* = 18)	G2 (*n* = 18)	G3 (*n* = 18)	G4 (*n* = 18)
	Mean ± SD^c^	Mean ± SD	Mean ± SD	Mean ± SD
Healthy nutrition diet	707.37 ± 180.78	837.06 ± 269.77	961.67 ± 205.76	914.72 ± 203.55
Carbohydrate-rich diet	668.16 ± 162.00	763.82 ± 223.66	947.50 ± 182.61	922.50 ± 186.57
Protein-rich diet	754.21 ± 117.95	845.00 ± 202.69	997.50 ± 136.90	964.44 ± 146.20
F	77.886	31.520	22.117	15.170
p^a^	0.001*	0.001*	0.001*	0.001*
1–2.week p^b^	0.037*	0.001*	1.000	1.000
1–3.week p^b^	0.370	1.000	1.000	0.382
2–3.week p^b^	0.006*	0.001*	0.281	0.670

G1: Mothers aged 24–29 in the 13–24 weeks postpartum period, G2: Mothers aged 30–35 in the 13–24 weeks postpartum period, G3: Mothers aged 24–29 in the 4–12 weeks postpartum period, G4: Mothers aged 30–35 in the 4–12 weeks postpartum period.

^a^Repeated Measures Analysis of Variance.

^b^Bonferroni test.

^c^*SD* Standard Deviation.

*p < 0.05.

The increase in glutamic acid, serine, glycine, histidine, tyrosine, valine, isoleucine, leucine, and lysine levels after the second week compared to the first week was statistically significant (*p* < 0.05). Compared to the values measured in the first week, there was an increase in aspartic acid, glutamic acid, glycine, and lysine levels, while serine, proline, methionine, and leucine levels decreased after the third week (*p* < 0.05). Compared with the second week, a decrease in serine, arginine, threonine, tyrosine, methionine, isoleucine, phenylalanine, and tryptophan amino acids was observed after the third week (*p* < 0.05). Regarding human milk, lactose decreased after the second week compared to the first week but increased after the third week compared to the first and second weeks (*p* < 0.05). Compared to the first week, riboflavin decreased in the second and third weeks (*p* < 0.05) (Table 2).

**Table 2:** Evaluation of changes in human milk amino acid, lactose, and riboflavin content based on weekly diets.

Variables (…/100 ml)	Diets			*χ2*	*p*^*1*^
	Healthy nutrition diet	Carbohydrate-rich diet	Protein-rich diet		
Aspartic acid (mg)	27.2(12.5–50.4)^a^	34.5(11.9–57.0)^ab^	40.2(17.6–46.0)^b^	7.500	0.024
Glutamic acid (mg)	120.6(38.2–142.7)^a^	130.8(82.1–159.4)^b^	129.7(96.6–136.4)^b^	22.500	<0.001
Serine (mg)	57.3(36.4–88.7)^a^	64.9(50.9–75.1)^b^	55.7(49.9–67.2)^c^	30.000	<0.001
Glycine (mg)	19.4(16.5–23.6)^a^	22.0(18.9–24.0)^b^	20.6(19.3–24.0)^b^	22.500	<0.001
Histidine (mg)	10.7(2.0–22.7)^a^	18.9(5.4–31.4)^b^	17.4(4.0–25.9)^ab^	7.500	0.024
Arginine (mg)	11.9(7.0–14.3)^ab^	11.9(7.2–23.7)^a^	11.4(8.0–18.7)^b^	14.000	0.001
Threonine (mg)	119.3(48.8–160.9)^ab^	123.8(83.8–154.3)^a^	122.4(92.3–138.7)^b^	7.500	0.024
Alanine (mg)	28.3(19.0–32.4)	28.5(23.4–33.1)	26.7(24.8–29.8)	6.000	0.050
Proline (mg)	86.8(77.4–97.4)^a^	84.3(75.2–94.7)^ab^	80.1(75.9–85.7)^b^	7.500	0.024
Tyrosine (mg)	25.5(13.2–29.3)^a^	27.2(19.9–32.7)^b^	25.4(22.6–29.1)^a^	22.500	<0.001
Valine (mg)	35.1(27.1–41.3)^a^	37.8(29.4–42.4)^b^	35.0(34.1–38.7)^ab^	7.500	0.024
Methionine (mg)	10.6(3.7–20.0)^a^	11.5(5.9–12.5)^a^	8.4(6.3–12.7)^b^	22.500	<0.001
Isoleucine (mg)	31.9(23.6–44.9)^a^	33.5(29.6–36.7)^b^	30.1(29.0–34.5)^a^	7.500	0.024
Leucine (mg)	64.2(40.0–77.9)^a^	66.7(52.7–76.6)^b^	62.4(55.3–65.4)^b^	7.500	0.024
Phenylalanine (mg)	28.3(19.4–34.3)^ab^	28.8(25.1–33.8)^a^	26.4(25.5–30.3)^b^	7.500	0.024
Lysine (mg)	44.6(15.7–64.3)^a^	48.7(25.9–85.3)^b^	49.7(26.3–61.5)^b^	22.500	<0.001
Tryptophan (mg)	9.9(9.2–11.0)^ab^	10.9(8.3–11.5)^a^	9.7(9.5–10.0)^b^	7.500	0.024
Lactose (g)	4.4(4.0–5.5)^a^	4.3(4.1–5.0)^b^	4.5(4.2–5.2)^c^	67.500	<0.001
Riboflavin (µg)	86.2(78.2–89.8)^a^	75.7(74.1–81.4)^b^	77.0(70.2–82.9)^b^	52.500	<0.001

^1^Friedman Test, ^a, b, c^ Dunn–Bonferroni.

There was no statistically significant relationship between the weekly body weight, BMI, fat mass, muscle mass, waist circumference, waist-to-hip ratio measurements of the mothers and the milk volume (*p* > 0.05) (Table 3). Additionally, there was no statistically significant relationship between the mothers’ height, body weight, BMI, fat mass, muscle mass, waist circumference, waist-to-hip ratio measurements, and milk composition (*p* > 0.05). However, a negative correlation (*r* = −0.270, *p* = 0.037) was found between the mother’s body weight in the third week and the amino acid content of the milk (Table 4).

**Table 3:** Comparison of the relationships between mothers’ weekly milk quantity and weekly anthropometric measurements within groups.

Anthropometric Measurements		Healthy nutrition diet				Carbohydrate-rich diet				Protein-rich diet			
		G1	G2	G3	G4	G1	G2	G3	G4	G1	G2	G3	G4
Body weight (kg)	*r*	0.132	−0.138	−0.050	0.042	0.221	−0.056	−0.141	0.172	−0.043	−0.074	−0.028	−0.017
	*p*	0.590	0.598	0.845	0.868	0.363	0.831	0.577	0.496	0.860	0.776	0.913	0.946
BMI (kg/m^2^)^1^	*r*	0.014	−0.252	−0.076	−0.064	0.093	−0.212	−0.188	0.070	−0.112	−0.214	−0.038	−0.170
	*p*	0.953	0.328	0.763	0.800	0.705	0.415	0.456	0.783	0.649	0.411	0.881	0.500
Fat mass (kg)	*r*	−0.004	−0.203	−0.046	−0.023	0.114	−0.136	−0.081	0.108	−0.188	−0.082	0.117	−0.043
	*p*	0.986	0.434	0855	0.927	0.642	0.603	0.748	0.670	0.440	0.754	0.643	0.867
Muscle mass (kg)	*r*	0.268	−0.066	−0.047	0.129	0.320	−0.015	−0.206	0.241	0.116	−0.058	−0.258	0.022
	*p*	0.268	0.802	0.854	0.610	0.181	0.953	0.411	0.336	0.636	0.825	0.301	0.932
Waist circumference (cm)	*r*	0.013	−0.189	−0.109	−0.019	0.132	−0.123	−0.138	0.122	−0.176	−0.084	−0.014	−0.033
	*p*	0.958	0.467	0.668	0.942	0.589	0.639	0.584	0.629	0.470	0.749	0.955	0.898
Waist-to-hip raito	*r*	−0.046	−0.216	−0.144	−0.031	0.047	−0.146	−0.159	0.078	−0.197	−0.114	−0.139	−0.083
	*p*	0.851	0.405	0.569	0.902	0.847	0.575	0.528	0.757	0.420	0.663	0.583	0.743

Pearson correlation analysis, ^1^BMI: Body Mass Index, G1: Mothers aged 24–29 in the 13–24 weeks postpartum period, G2: Mothers aged 30–35 in the 13–24 weeks postpartum period, G3: Mothers aged 24–29 in the 4–12 weeks postpartum period, G4: Mothers aged 30–35 in the 4–12 weeks postpartum period.

**Table 4:** Distribution of the relationships between participants’ weekly milk components and anthropometrics.

		Healthy nutrition diet			Carbohydrate-rich diet			Protein-rich diet		
		AA^a^	Lactose	B_2_	AA	Lactose	B_2_	AA	Lactose	B_2_
Mother’s height (cm)	*r*	−0.156	0.231	−0.231	0.091	0.144	−0.060	−0.177	0.231	0.231
	*p*	0.233	0.076	0.076	0.491	0.272	0.651	0.176	0.076	0.076
Mother’s weight (kg)	*r*	−0.067	0.198	−0.198	0.197	0.125	0.099	−0.270	0.195	0.195
	*p*	0.613	0.130	0.130	0.131	0.340	0.454	0.037*	0.135	0.135
BMI (kg/m^2^)^b^	*r*	−0.036	0.123	−0.123	0.160	0.067	0.111	−0.198	0.107	0.107
	*p*	0.784	0.350	0.350	0.223	0.610	0.396	0.129	0.417	0.417
Fat mass (kg)	*r*	−0.110	0.220	−0.220	0.159	0.117	0.037	−0.222	0.180	0.180
	*p*	0.286	0.124	0.124	0.426	0.594	0.906	0.237	0.359	0.359
Muscle mass (kg)	*r*	0.047	0.183	−0.183	0.225	0.169	0.167	−0.237	0.202	0.202
	*p*	0.719	0.163	0.163	0.084	0.196	0.203	0.068	0.121	0.121
Waist circumference (cm)	*r*	−0.102	0.209	−0.209	0.171	0.119	0.057	−0.237	0.183	0.183
	*p*	0.440	0.108	0.108	0.190	0.364	0.664	0.068	0.162	0.162
Waist-to-hip raito	*r*	−0.097	0.232	−0.232	0.194	0.144	0.071	−0.252	0.201	0.201
	*p*	0.460	0.074	0.074	0.138	0.271	0.590	0.052	0.124	0.124

Spearman’s rho correlation analysis.

^a^AA: Total analyzed amino acids.

^b^*BMI* Body Mass Index.

*p < 0.05.

## Discussion

Maternal nutrition is a crucial factor influencing both the volume of human milk [2] and its nutritional composition [27]. The impact of maternal nutrition on the macronutrient composition of human milk has been investigated by numerous researchers [28] with particular emphasis on studies focusing on the fatty acid composition of human milk [29]. This research was conducted because of the outdated nature and conflicting results of studies on the protein and lactose content of human milk, lack of current information, and absence of a nationally conducted study. A total of 72 mothers were enrolled in the study, categorized as follows: 24–29 years old in the lactation period of 13–24 weeks (*n* = 18), 30–35 years old in the lactation period of 13–24 weeks (*n* = 18), 24–29 years old in the lactation period of 4–12 weeks (*n* = 18), and 30–35 years old in the lactation period of 4–12 weeks (*n* = 18).

When examining the relationship between the amount of human milk and different nutritional programs, differences in milk quantity were observed between a protein-rich diet and a carbohydrate-rich diet. In lactating mothers aged 13–24 weeks, an increase in milk quantity was observed following the protein-rich diet. In a previous study, mothers who consumed a controlled diet with calculated protein and energy intake were found to have a positive correlation between milk production and the intake of lysine, leucine, and nitrogen [30]. Therefore, the present study aligns with findings in the literature.

When examining the relationship between the composition of human milk and different dietary programs, several amino acids (glutamic acid, serine, glycine, histidine, tyrosine, valine, isoleucine, leucine, and lysine) increased following the carbohydrate-rich diet compared with the healthy nutrition diet. These results appear to be consistent with those of a study conducted by Hascoët et al. [31] who demonstrated a positive correlation between maternal carbohydrate intake and the protein content of human milk. Moreover, in a study comparing the milk of Mexican women following a high-carbohydrate and low-fat diet with that of American women adhering to a high-fat and low-carbohydrate diet, it was observed that the milk of Mexican women exhibited higher concentrations of serine, proline, cysteine, methionine, and tryptophan [32]. As these studies were conducted in different regions with diverse populations and cultures, the results may vary.

In this study, the lactose content of milk decreased following the carbohydrate-rich diet compared with the healthy nutrition diet. Previous studies have shown that the lactose content of milk is not directly associated with maternal carbohydrate intake [33]. Similarly, Mohammad et al. [14] demonstrated that diets with varying amounts of carbohydrates and fats did not have a significant impact on lactose concentration in human milk. These findings suggest that the quantity of carbohydrates consumed does not directly lead to an increase in the lactose content of the human milk.

When comparing the healthy nutrition diet with the protein-rich diet, an increase was observed only in aspartic acid, glutamic acid, glycine, and lysine levels following the protein-rich diet. In contrast to the carbohydrate-rich diet, no increase in amino acid levels was observed after the protein-rich diet. A study conducted with breastfeeding mothers in Beijing, where mothers with lower protein intake had their milk analyzed, found no significant differences in 18 amino acids [34]. Conversely, a cross-over study in Sweden comparing a protein-rich diet with a low-protein diet reported a higher total protein content in the milk when consuming a protein-rich diet (8.83 g/day compared to 7.31 g/day) [13]. These results indicate that the amino acid composition of human milk may vary depending on the dietary pattern, and that different outcomes can be obtained in studies conducted in diverse regions, cultures, and socioeconomic environments.

Following the protein-rich diet, the lactose content of the milk was significantly higher than that at other weeks. Similar results were found in a meta-analysis conducted by Xi et al. [35]. In these studies, the observed differences following the protein-rich diet may be attributed to the increased protein utilization in the body due to metabolic processes. It is conceivable that as a result of a protein-rich diet, more proteins might have been utilized in the body, while the low carbohydrate intake in the diet could have led to a higher passage of carbohydrates into milk compared to proteins.

In the comparison of the riboflavin content of milk between the healthy nutrition diet and other dietary programs, both the carbohydrate-rich and protein-rich diets showed a decrease in riboflavin levels. No study with similar observations has been reported in the literature.

The quantity of milk showed no significant differences with mother’s age, body weight, BMI, fat tissue and muscle mass, waist circumference, and waist-to-hip ratio. While one study identified a negative relationship between milk quantity and the mother’s weight, BMI, lean body mass, and fat tissue [36] some studies did not find a significant relationship between milk volume and the mother’s age or anthropometric changes [37, 38]. Factors influencing milk quantity are not limited to these factors alone, and it is also necessary to examine other parameters.

Following the protein-rich diet, a negative relationship was observed between milk protein and mother’s body weight. However, in subsequent weeks, no significant relationship was found between the mother’s body weight and milk lactose, riboflavin, and amino acid content (*p* > 0.05). A study comparing breastfeeding mothers who were obese and non-obese found that the fat content and sometimes the protein content of milk were higher in obese mothers [39]. The reason for the mothers’ weight gain may be an increase in carbohydrate and fat intake, coupled with a decrease in protein intake. This situation may have led to a decrease in the proportion of proteins transferred to the milk.

The scope of this study is limited to mothers residing in a specific district of Istanbul; thus, it reflects the sociocultural and socioeconomic characteristics unique to this region. During the analysis of milk samples, challenges were encountered, such as the inability to use laboratory equipment for an extended period and the high cost of materials, all of which were covered by the researcher. The absence of financial support was also a limitation of this study.

## Conclusion

Human milk is a unique and crucial component of infant nutrition. In this study of the quantity and composition of human milk based on nutritional programs, it was found that a diet rich in carbohydrates led to an increased presence of amino acid components in the milk, whereas a protein-rich diet resulted in an increase in both the quantity of milk and the lactose component. These findings suggest that dietary composition plays a significant role in influencing the amino acid and lactose contents of human milk. Nevertheless, it would be appropriate to replicate this study in a broader population by using more advanced techniques.

## Data Availability

The data analyzed in the current study are not publicly available due to data confidentiality reasons. Data analyzed in this study are available from the corresponding author upon reasonable request.
